# Common characteristics of variants linked to autism spectrum disorder in the WAVE regulatory complex

**DOI:** 10.3389/fncom.2025.1704350

**Published:** 2025-11-12

**Authors:** Song Xie, Ke Zuo, Silvia De Rubeis, Giorgio Bonollo, Giorgio Colombo, Paolo Ruggerone, Paolo Carloni

**Affiliations:** 1 Computational Biomedicine, Institute of Neuroscience and Medicine INM-9, Forschungszentrum Jülich GmbH, Jülich, Germany; 2 Department of Physics, RWTH Aachen University, Aachen, Germany; 3 Chongqing Key Laboratory of Kinase Modulators as Innovative Medicine, National & Local Joint Engineering Research Center of Targeted and Innovative Therapeutics, College of Pharmacy (International Academy of Targeted Therapeutics and Innovation), Chongqing University of Arts and Sciences, Chongqing, China; 4 Department of Physics, University of Cagliari, Cagliari, Italy; 5 Seaver Autism Center for Research and Treatment, Icahn School of Medicine at Mount Sinai, New York, NY, United States; 6 Department of Psychiatry, Icahn School of Medicine at Mount Sinai, New York, NY, United States; 7 Icahn School of Medicine at Mount Sinai, The Mindich Child Health and Development Institute, New York, NY, United States; 8 Icahn School of Medicine at Mount Sinai, Friedman Brain Institute, New York, NY, United States; 9 Department of Pharmacological Sciences, Icahn School of Medicine at Mount Sinai, New York, NY, United States; 10 Alper Center for Neural Development and Regeneration, Icahn School of Medicine at Mount Sinai, Friedman Brain Institute, New York, NY, United States; 11 Dipartimento di Chimica, Università di Pavia, Pavia, Italy; 12 JARA Institute: Molecular Neuroscience and Imaging, Institute of Neuroscience and Medicine INM-11, Forschungszentrum Jülich GmbH, Jülich, Germany

**Keywords:** WAVE regulatory complex, neurodevelopmental disorder, autism spectrum disorder, missense variants, molecular dynamics, allosteric analysis

## Abstract

Six variants associated with autism spectrum disorder (ASD) abnormally activate the WASP-family Verprolin-homologous protein (WAVE) regulatory complex (WRC), a critical regulator of actin dynamics. This abnormal activation may contribute to the pathogenesis of this disorder. Using molecular dynamics (MD) simulations, we recently investigated the structural dynamics of wild-type (WT) WRC and R87C, A455P, and Q725R WRC disease-linked variants. Here, by extending MD simulations to I664M, E665K, and D724H WRC, we suggest that *all* of the mutations weaken the interactions and affect intra-complex allosteric communication between the WAVE1 active C-terminal region (ACR) and the rest of the complex. This might contribute to an abnormal complex activation, a hallmark of WRC-linked ASD. In addition, all mutants but I664M destabilize the ACR V-helix and increase the participation of ACR in large-scale movements. All these features may also abnormally influence the inactive WRC toward a dysfunctional state. We hypothesize that small-molecule ligands counteracting these effects may help restore normal WRC regulation in ASD-related variants.

## 1 Introduction

**Figure 1:**
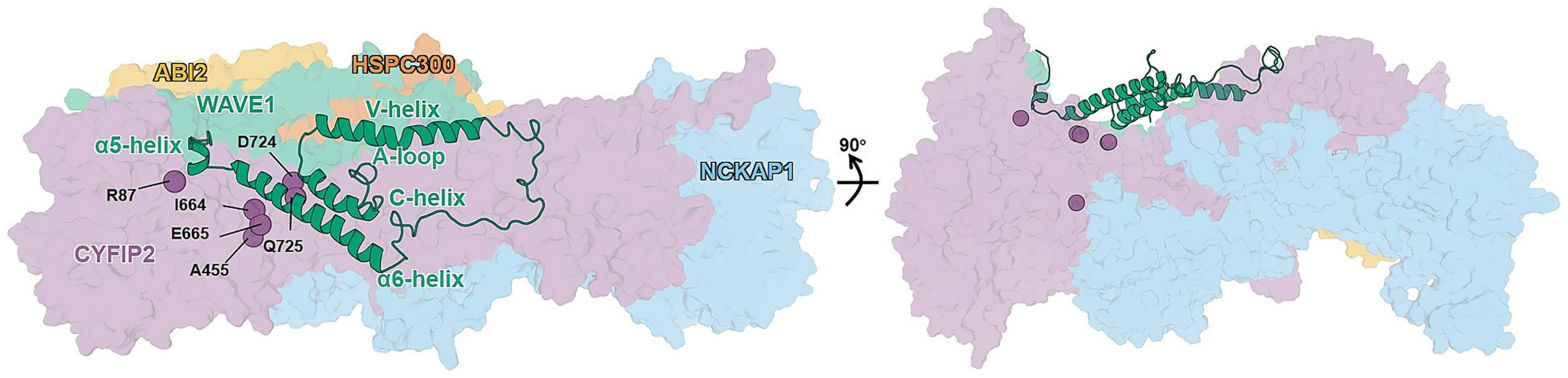
Architecture of WT WRC inactive form. The subunits (CYFIP2, purple; NCKAP1, blue; WAVE1, green; HSPC300, burnt orange; and ABI2, yellow) are shown as surfaces except for ACR (cartoon), which consists of α5, α6, V-, and C-helices, A-loop, and connecting loops. The mutation sites discussed in the text are shown as labeled spheres. The right-hand panel shows the 90° rotated view of the complex, emphasizing the buried location of the A455. The model is taken from our previous work (Xie et al., 2025b).

Autism spectrum disorder (ASD) is one of the most common neurodevelopmental disorders (NDDs) in childhood, affecting approximately 1% of the population (Zeidan et al., 2022). Individuals with ASD are enriched in *de novo* missense variants that disrupt protein–protein interactions (PPIs), with estimates that up to 25% of PPIs are disrupted, and genes encoding proteins involved in disrupted PPIs are correspondingly more readily identifiable as ASD risk genes (Chen et al., 2018, 2020). One such gene (Fu et al., 2022; Xie et al., 2025a, 2025b), *CYFIP2* (cytoplasmic FMR1-interacting protein 2), encodes a subunit of the WAVE (WASP family verprolin-homologous) regulatory complex (WRC) (Chen et al., 2010) (Figure 1). This large hetero-pentameric complex comprises an elongated, pseudo-symmetrical CYFIP1/2-NCKAP1 (non-catalytic region of tyrosine Kinase Associated Protein 1) dimer and a trimer of ABI1/2/3 (Abelson interactor 1/2/3), HSPC300 (hematopoietic stem/progenitor cell protein 300), and WAVE1/2/3 proteins (Chen et al., 2010) (Figure 1). Activation of WRC has been demonstrated to regulate actin remodeling (Rottner et al., 2021), a process that is critical for brain function and development, such as synapse maturation and formation (De Rubeis et al., 2013; Davenport et al., 2019). Under physiological conditions, WRC remains “inactive” until it binds to cellular partners such as the GTPase Rac1 (Chen et al., 2017). The latter activates the complex by releasing the WAVE1 active C-terminal region (ACR) without apparently affecting the rest of the complex (Figure 1) (Ding et al., 2022).

Six CYFIP2 variants related to ASD (R87C, A455P, I664M, E665K, D724H, and Q725R) abnormally convert WRC from an “inactive” state to an “active-like” state even when there is no binding to cellular partners (Figure 1) (Schaks et al., 2020)^1^. This abnormal activation can alter the balance between excitatory and inhibitory, spine morphology, and neuronal excitability, thus increasing the risk of ASD and other NDDs (De Rubeis et al., 2013; Nakashima et al., 2018; Zweier et al., 2019; Zhao and Guan, 2024). Previous all-atom molecular dynamics (MD) studies from us have shown that an internal variant (A455P) and two variants of the ACR/CYFIP2 interface (R87C and Q725R) similarly reduced ACR interactions with the rest of the complex, although located in different regions (Figure 1) (Xie et al., 2025b).

To complete this investigation, here we conducted the same MD protocol on the WRC carrying the remaining CYFIP2 variants (I664M, E665K, and D724H). We then compared the results of all six variants to comprehensively study the impact of ASD-associated variants on PPIs and structural dynamics of the WRC. This might provide mechanistic insights into ASD-associated WRC dysfunctions and a rational basis for therapies that restore normal WRC regulation.

## 2 Results

For each system, the final 1.5-μs equilibrated trajectories from each of the three independent replicates were pooled for analysis, yielding a total of 4.5-μs trajectories (more details in the Section Materials and Methods and in Supplementary material). In no case was global unfolding observed (Supplementary Table S1 and Supplementary Figures S1–S4)^2^.

### 2.1 Positions subject to mutations

**Figure 2:**
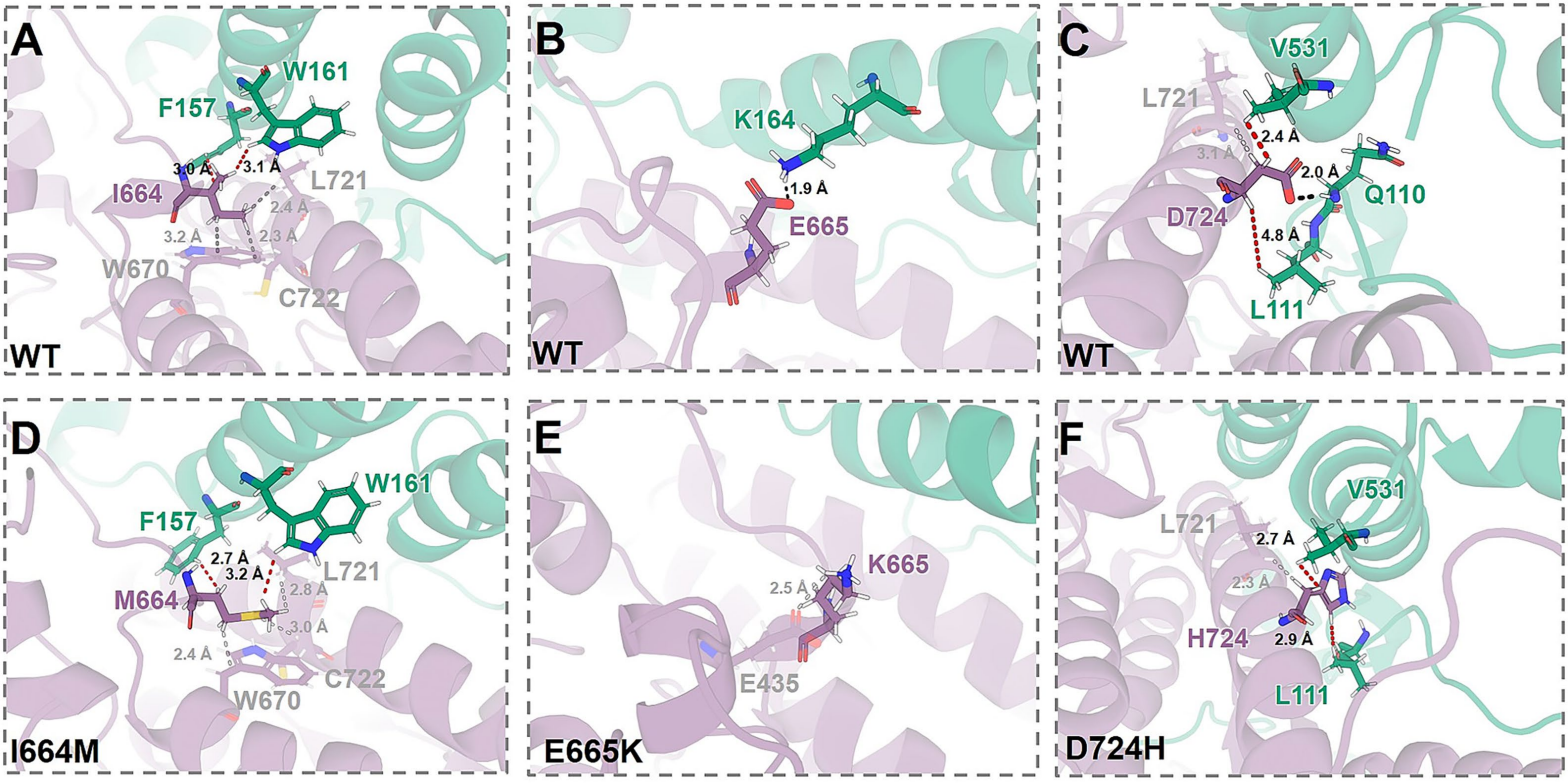
Alterations associated with I664M **(A, D)**, E665K **(B, E)**, and D724H **(C, F)** in the chemical environments at the mutation sites. The structures of WT WRC, obtained from our previous work (Xie et al., 2025b), and of the variants from MD simulations (see Methods in Supplementary material). CYFIP2 and WAVE1 are depicted as cartoons, colored purple and green, respectively. Mutated residues and contacting groups are represented as sticks. Hydrogen bonds and the shortest van der Waals contact are indicated by black and red dashed lines, respectively.

In the WT complex, the residues of the mutant site are involved in a series of interactions between subunits: (i) I664 (CYFIP2) forms van der Waals contacts with F157 and W161 (both in ACR), at times of 99 and 94%, respectively (Figure 2A and Supplementary Table S2). (ii) E665 (CYFIP2) forms a salt bridge with K164 (ACR) with an occupancy of 53% (Figure 2B and Supplementary Table S2). (iii) D724 (CYFIP2) forms a hydrogen bond with Q110 (WAVE1 outside the ACR) with 27% occupancy, and van der Waals contacts with V531 (ACR) and L111 (WAVE1 outside the ACR), at times of 100 and 50% (Figure 2C and Supplementary Table S2). The variants (I664M, E665K, and D724H) retain van der Waals contacts but disrupt hydrogen bonds and salt bridges between subunits (Figures 2D–F and Supplementary Table S2).

### 2.2 ACR/WRC interface contacts

**Figure 3:**
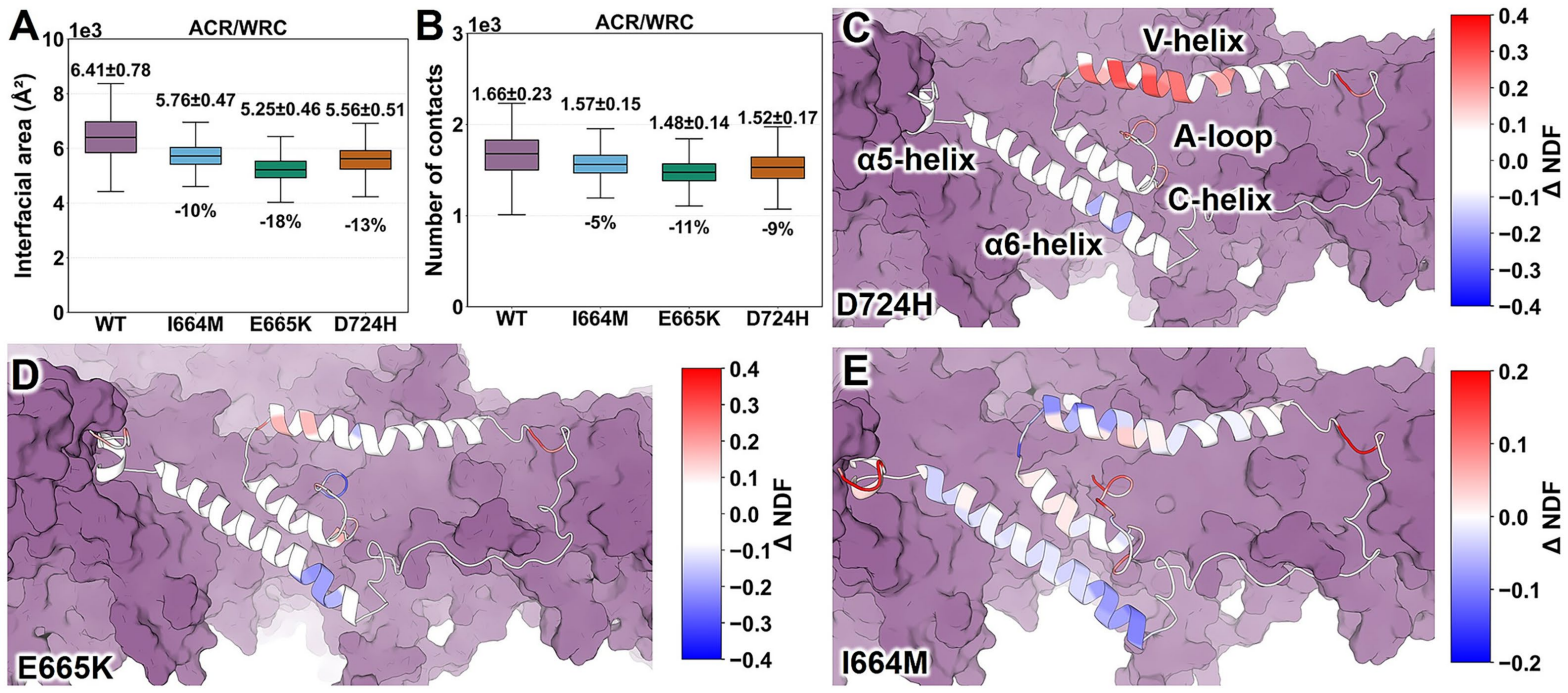
Impact of I664M, E665K, and D724H mutations on interactions between ACR and the rest of the WRC. **(A)** ACR/WRC interface areas. **(B)** Number of contacts with heavy atoms within 5 Å. **(C–E)** *ΔNDF_i_* values for ACR residues. They range from −0.4 (blue) to +0.4 (red) for D724H and E665K and from −0.2 (blue) to +0.2 (red) for I664M. ACR is represented as a cartoon, while the rest is depicted as a purple surface. *ΔNDF_i_* data for the entire complex are shown in Supplementary Figure S6. Data for WT WRC were obtained from our previous work (Xie et al., 2025b).

Three variants similarly reduce ACR/WRC interactions [by 10 to 18% for the interface area; from 5 to 11% in the number of contacts (*Nc*)] compared to the WT complex (Figures 3A,B), while changes to other interfaces are smaller in comparison (interface area between −13 and −2% and *Nc* between −10 and +6%; Supplementary Figure S5). Thus, all three variants reduce the stability of the ACR/WRC interface (Figures 3A,B). However, they do not affect the overall stability of the complex: Supplementary Figure S4 shows that the changes of radius of gyration in variants range from −0.1 to 0.1% relative to the WT complex.

### 2.3 ACR local disorder

The normalized distance fluctuation for residue *i* (*NDF_𝑖_*) quantifies how the *i-*th residue moves in coordination with the rest of the WRC (Morra et al., 2012). Positive values of the differences in *NDF_𝑖_* values (*ΔNDF_i_*; variant minus WT) indicate an increase in local disorder, while negative values suggest the opposite (Morra et al., 2014; Triveri et al., 2023; Castelli et al., 2024; Frasnetti et al., 2024; Torielli et al., 2025). Minor changes are observed in most ACR residues in the loops, α5, and C-helices (*|ΔNDF_𝑖_* | ≤ 0.1; Figures 3C–E). D724H and E665K (Figures 3C,D) increase the propensity for uncoiling of the V-helix segments (*ΔNDF_𝑖_* ranges from 0.12 to 0.30) compared to the WT complex, with only one exception (E515; *ΔNDF_𝑖_* = − 0.12) in E665K. In these two variants, some regions of the α6 helix become more rigid. I664M exerts a weaker effect than other variants (Figure 3E) (Xie et al., 2025b): it destabilizes the α5 helix, stabilizes the α6 helix, and has a mixed impact (both stabilizing and destabilizing) on the V-helix (Figure 3E). Complex and variant-dependent changes are observed in loop regions (Figures 3C–E). The rest of the WRC is not significantly affected (Supplementary Figure S6). Our results suggest that E665K and D724H commonly destabilize the V-helix (Figures 3C,D), while I664M exerts a mild and mixed (both stabilizing and destabilizing) effect (Figure 3E).

### 2.4 Large-scale movements

Dynamic cross-correlation analysis shows that three variants alter the correlations between residue motions within the WRC, with moderate consistency (Supplementary Figure S7). Pairwise comparisons of variant-induced changes in motion correlation matrices yield cosine similarities ranging from 0.43 to 0.63 (Supplementary Table S3) and Spearman correlations ranging from 0.38 to 0.60 (Supplementary Table S4). Principal component analysis (PCA) reveals that the three largest eigenvectors (PC1–PC3) collectively account for approximately 50% of the total variance (Supplementary Figure S8). PC1–PC3 of E665K and D724H variants feature an increase in ACR contributions relative to the WT complex (from 20 to 42 and 25%, respectively), while I664M shows a smaller decrease (15%)^3^. These results suggest that E665K and D724H variants may promote ACR detachment by increasing its participation in large-scale movements, whereas I664M does not.

### 2.5 Allosteric analysis

**Figure 4:**
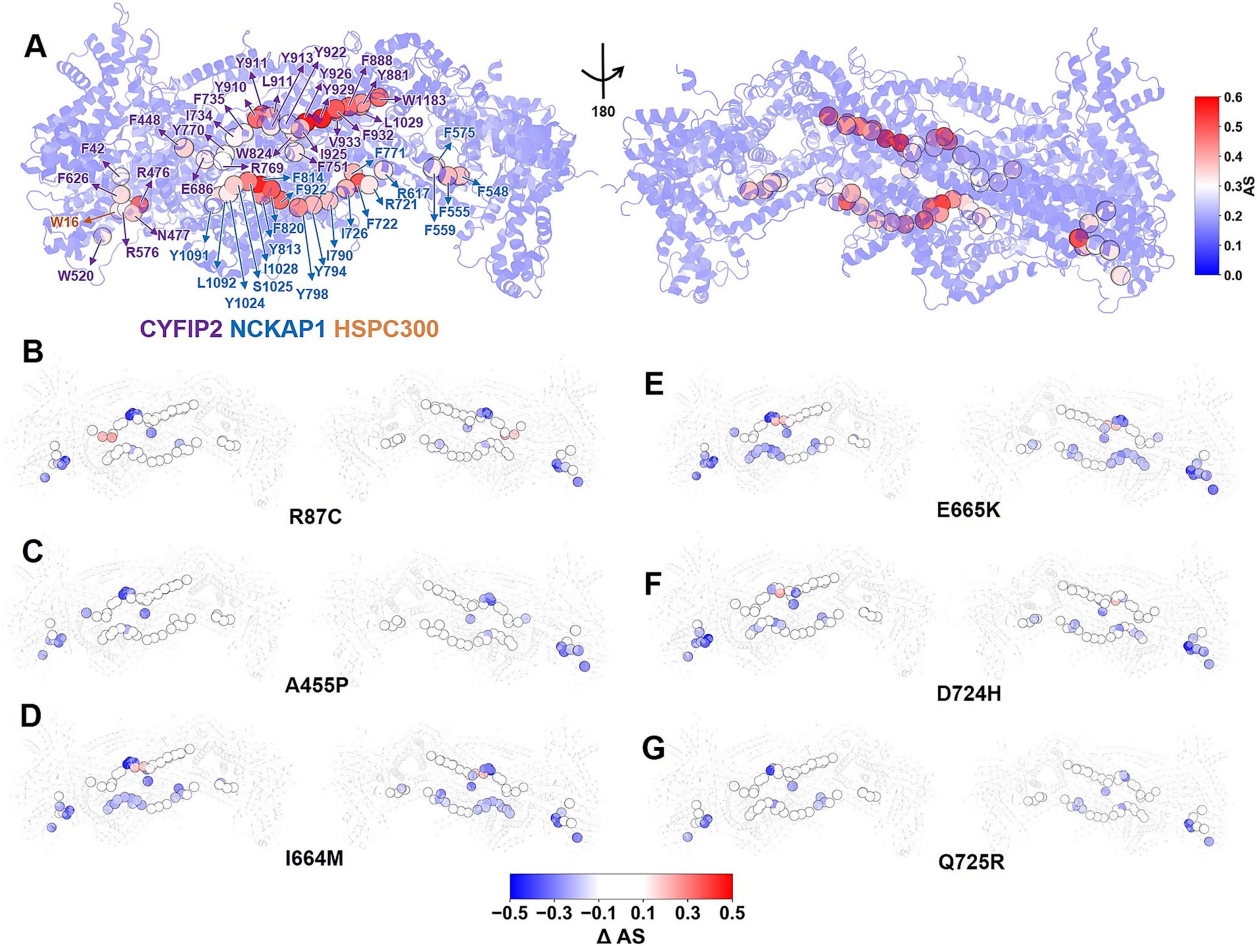
Effect of the six ASD-linked variants on allosteric communication within the complex. **(A)** Allosteric scores (*AS_i_*) for WT WRC residues, ranging from 0 (blue) to 0.6 (red). Residues with *AS_i_* greater than 0.3 are designated as allosteric hubs, illustrated by spheres. The residue index of allosteric hubs is labeled as follows: purple for CYFIP2, blue for NCKAP1, and orange for HSPC300. **(B–G)** *ΔAS_i_* for allosteric hubs in variants, ranging from −0.5 (blue) to 0.5 (red). The WRC is shown as a cartoon. The suppression of allosteric hubs in all variants indicates that all variants, not only A455P, weaken ACR/WRC interactions via allosteric changes.

Here, we calculate the allosteric score to quantify the contribution of each residue *i* to the long-range communication within the complex (*AS_i_*; see details in Supplementary material) (Schneider and Antes, 2022). The difference between the scores of the variant and those of the WT, *ΔAS_i_* (variant minus WT), reflects the impact of the variant on the allosteric pathways within the complex. A positive *ΔAS_i_* indicates greater allosteric importance. Negative values indicate the opposite effect (Schneider and Antes, 2022). In the WT complex, we identify 50 allosteric hubs (*AS_i_* equal to or greater than half of the maximum, 0.3): 28 in CYFIP2, 21 in NCKAP1, and 1 in HSPC300 (Figure 4A). Notably, all variants disrupt allosteric communication within the complex (Figures 4B–G): 10, 11, 22, 25, 15, and 10 allosteric hubs show reduced allosteric importance (*ΔAS_i_* less than −0.1) in variants R87C, A455P, I664M, E665K, D724H, and Q725R, respectively. In contrast, few hubs show an increased role (*ΔAS_i_* greater than 0.1): 2, 0, 2, 2, 1, and 0 for the R87C, A455P, I664M, E665K, D724H, and Q725R variants, respectively. The remaining hubs showed only minor variations (*|ΔAS_i_|* equal to or less than 0.1). The common erosion of the allosteric role in hubs suggests that all variants disrupt the long-range communication between the ACR and the rest of the WRC (Figure 4). This implies that not only A455P (Xie et al., 2025b), but all six variants could decrease ACR/WRC interactions via allosteric changes.

## 3 Discussion

Disruption of ACR’s V-helix contacts with the rest of the complex abnormally activates the WRC (Chen et al., 2010). Here, we used molecular dynamics simulations to examine how ASD-associated variants influence the structural dynamics of ACR, particularly those of the V-helix. All mutations are located in CYFIP2: five are at the interface with ACR, and one (A455P) is buried internally (Figure 1). Our main findings are as follows (Supplementary Table S5)^4^:

1. None of the variants show global unfolding or loss of complex stability (Supplementary Figures S4,S5).
2. All of the mutations weaken ACR/WRC contacts relative to the WT complex, regardless of their location or chemical properties (Figures 1, 3A,B). The mutations disrupt long-range communication between the ACR (including its V-helix) and the rest of the WRC (Figure 4), which may promote aberrant ACR detachment and WRC activation. A455P does so through allosteric effects (Figure 4C), while the others do so by disrupting interfacial hydrogen bonds and/or salt bridges at their respective sites (Figure 2 and Supplementary Table S2). These findings are consistent with the ~50% reduction in CYFIP2 binding to ACR’s V-helix, C-helix, and A-loop for R87C WRC (Nakashima et al., 2018).
3. Most variants increase their propensity for unwinding in the V-helix relative to WT (Figures 3C,D). This may indicate a process leading to ACR detachment (Chen et al., 2010). However, I664M exerts a weaker mixed effect (both stabilizing and destabilizing; see Figure 3E). Additionally, I664M decreases ACR participation in large-scale movements compared to WT, while the others show an opposite trend.

In summary, we propose that ASD-linked mutations facilitate ACR detachment (particularly the V-helix) by weakening its contacts with the rest of the complex and eroding long-range allosteric communication within the complex. Additionally, all but I664M may favor ACR detachment by increasing V-helix disorder in ACR and enlarging ACR participation in large-scale movements. The present findings are consistent with those of previous *in vivo* experiments, which demonstrated that these six mutations cause aberrant lamellipodia without the binding of their cellular partners (Schaks et al., 2020). The lamellipodia are cellular hallmarks of ACR detachment and WRC activation (Schaks et al., 2018).

Unfortunately, no ligand or therapeutic strategy exists yet for the WRC dysfunction associated with ASD-linked mutations. In an effort at identifying new therapeutic agents counteracting the deranged effect of the disease, we hypothesize here that ligands stabilizing the ACR/WRC interface or reconstructing allosteric communication hold the potential to restore normal WRC regulation in these ASD-linked variants. The ACR/CYFIP2 interface is a promising target region for ligands that stabilize ACR/WRC interactions, since CYFIP2 is the primary interactor within the WRC (Figure 1). *In vivo* studies could determine whether such ligand candidates restore the function of the WT, namely the formation of lamellipodia only after Rac1 binding (Schaks et al., 2020).

## 4 Materials and methods

Details of model construction and property calculations are described in the Supplementary material.

### 4.1 Molecular dynamics simulation

MD simulations were performed using AMBER 22 software (Case et al., 2005). Long-range electrostatic interactions were calculated using the Particle Mesh Ewald (PME) method (Darden et al., 1993). A cutoff distance of 10 Å was applied to short-range non-bonded interactions, which include Lennard-Jones forces and the short-range component of the PME calculation. Periodic boundary conditions were applied. The systems underwent three successive minimization cycles: (i) 10,000 steep descent steps followed by 10,000 conjugate gradient minimization steps with a 100 kcal/(mol·Å^2^) constraint applied to the entire solute; (ii) the same protocol with the same constraints, but applied only to heavy atoms; and (iii) the same protocol without any constraints. The systems were then heated from 100 K to 310 K in 0.5 ns using Langevin dynamics (Lemons and Gythiel, 1997). A constraint of 100 kcal/(mol·Å^2^) was applied to the heavy atoms. The systems were then subjected to another 0.5 ns at 310 K without restrictions. An integration time interval of 1 fs was used during heating. Subsequently, each system was subjected to three independent isobaric-isothermal (NPT) simulations lasting 2 μs, each started at different velocities. The temperature (310 K) and pressure (1 atm) were maintained using Langevin dynamics (Lemons and Gythiel, 1997) and a Monte Carlo barostat (Åqvist et al., 2004), respectively. A time step of 2 fs was used during NPT simulations. The trajectories were output at a frequency of 10 ps. The data for the MD simulations, including the input files, parameter files, and analysis scripts, can be found in the Zenodo repository: https://zenodo.org/record/15481836.

## Data Availability

The datasets presented in this study can be found in online repositories. The names of the repository/repositories and accession number(s) can be found in the article/Supplementary material.

## Footnotes

^1^Other ASD-linked variants have been summarized in our previous work (Xie et al., 2025a). Y108H in CYFIP2 increases Rac1 binding abnormally (Schaks et al., 2020), but it is unclear if the remaining variants also promote aberrant WRC activation.

^2^The WT, R87C, A455P, and Q725R WRC trajectories for analysis were taken from our previous work (Xie et al., 2025b).

^3^The convergence of these results is affected by the timescale of the simulations (6 μs). Consequently, we restrict our discussion to qualitative trends.

^4^Except for the allosteric analysis, the findings of WT, R87C, A455P, and Q725R WRC discussed here were obtained from our previous work (Xie et al., 2025b).

## References

[ref1] Åqvist J. Wennerström P. Nervall M. Bjelic S. Brandsdal B. (2004). Molecular dynamics simulations of water and biomolecules with a Monte Carlo constant pressure algorithm. Chem. Phys. Lett. 384, 288–294. doi: 10.1016/j.cplett.2003.12.039

[ref2] Case D. A. Cheatham T. E. III Darden T. Gohlke H. Luo R. Merz K. M. Jr. . (2005). The amber biomolecular simulation programs. J. Comput. Chem. 26, 1668–1688. doi: 10.1002/jcc.20290, PMID: 16200636 PMC1989667

[ref3] Castelli M. Magni A. Bonollo G. Pavoni S. Frigerio F. Oliveira A. S. F. . (2024). Molecular mechanisms of chaperone-directed protein folding: insights from atomistic simulations. Protein Sci. 33:e4880. doi: 10.1002/pro.4880, PMID: 38145386 PMC10895457

[ref4] Chen Z. Borek D. Padrick S. B. Gomez T. S. Metlagel Z. Ismail A. M. . (2010). Structure and control of the actin regulatory WAVE complex. Nature 468, 533–538. doi: 10.1038/nature09623, PMID: 21107423 PMC3085272

[ref5] Chen B. Chou H. Brautigam C. A. Xing W. Yang S. Henry L. . (2017). Rac1 GTPase activates the WAVE regulatory complex through two distinct binding sites. eLife 6:e29795. doi: 10.7554/eLife.29795, PMID: 28949297 PMC5614565

[ref6] Chen S. Fragoza R. Klei L. Liu Y. Wang J. Roeder K. . (2018). An interactome perturbation framework prioritizes damaging missense mutations for developmental disorders. Nat. Genet. 50, 1032–1040. doi: 10.1038/s41588-018-0130-z, PMID: 29892012 PMC6314957

[ref7] Chen S. Wang J. Cicek E. Roeder K. Yu H. Devlin B. (2020). De novo missense variants disrupting protein–protein interactions affect risk for autism through gene co-expression and protein networks in neuronal cell types. Mol. Autism. 11:76. doi: 10.1186/s13229-020-00386-7, PMID: 33032641 PMC7545940

[ref8] Darden T. York D. Pedersen L. (1993). Particle mesh Ewald: an N·log(N) method for Ewald sums in large systems. J. Chem. Phys. 98, 10089–10092. doi: 10.1063/1.464397

[ref9] Davenport E. C. Szulc B. R. Drew J. Taylor J. Morgan T. Higgs N. F. . (2019). Autism and schizophrenia-associated CYFIP1 regulates the balance of synaptic excitation and inhibition. Cell Rep. 26, 2037–51.e6. doi: 10.1016/j.celrep.2019.01.092, PMID: 30784587 PMC6381785

[ref10] De Rubeis S. Pasciuto E. Li K. Fernández E. Di Marino D. Buzzi A. . (2013). CYFIP1 coordinates mRNA translation and cytoskeleton remodeling to ensure proper dendritic spine formation. Neuron 79, 1169–1182. doi: 10.1016/j.neuron.2013.06.039, PMID: 24050404 PMC3781321

[ref11] Ding B. Yang S. Schaks M. Liu Y. Brown A. J. Rottner K. . (2022). Structures reveal a key mechanism of WAVE regulatory complex activation by Rac1 GTPase. Nat. Commun. 13:5444. doi: 10.1038/s41467-022-33174-3, PMID: 36114192 PMC9481577

[ref12] Frasnetti E. Cucchi I. Pavoni S. Frigerio F. Cinquini F. Serapian S. A. . (2024). Integrating molecular dynamics and machine learning algorithms to predict the functional profile of kinase ligands. J. Chem. Theory Comput. 20, 9209–9229. doi: 10.1021/acs.jctc.4c01097, PMID: 39387368

[ref13] Fu J. M. Satterstrom F. K. Peng M. Brand H. Collins R. L. Dong S. . (2022). Rare coding variation provides insight into the genetic architecture and phenotypic context of autism. Nat. Genet. 54, 1320–1331. doi: 10.1038/s41588-022-01104-0, PMID: 35982160 PMC9653013

[ref14] Lemons D. S. Gythiel A. (1997). Paul Langevin’s 1908 paper “on the theory of brownian motion” [“Sur la théorie du mouvement brownien,” C. R. Acad. Sci. (Paris) 146, 530–533 (1908)]. Am. J. Phys. 65, 1079–1081. doi: 10.1119/1.18725

[ref15] Morra G. Genoni A. Colombo G. (2014). Mechanisms of differential allosteric modulation in homologous proteins: insights from the analysis of internal dynamics and energetics of PDZ domains. J. Chem. Theory Comput. 10, 5677–5689. doi: 10.1021/ct500326g, PMID: 26583250

[ref16] Morra G. Potestio R. Micheletti C. Colombo G. (2012). Corresponding functional dynamics across the Hsp90 chaperone family: insights from a multiscale analysis of MD simulations. PLoS Comput. Biol. 8:e1002433. doi: 10.1371/journal.pcbi.1002433, PMID: 22457611 PMC3310708

[ref17] Nakashima M. Kato M. Aoto K. Shiina M. Belal H. Mukaida S. . (2018). De novo hotspot variants in CYFIP2 cause early-onset epileptic encephalopathy. Ann. Neurol. 83, 794–806. doi: 10.1002/ana.25208, PMID: 29534297

[ref18] Rottner K. Stradal T. E. B. Chen B. (2021). WAVE regulatory complex. Curr. Biol. 31, R512–R517. doi: 10.1016/j.cub.2021.01.086, PMID: 34033782 PMC8882368

[ref19] Schaks M. Reinke M. Witke W. Rottner K. (2020). Molecular dissection of neurodevelopmental disorder-causing mutations in CYFIP2. Cells 9:1355. doi: 10.3390/cells9061355, PMID: 32486060 PMC7348743

[ref20] Schaks M. Singh S. P. Kage F. Thomason P. Klünemann T. Steffen A. . (2018). Distinct interaction sites of Rac GTPase with WAVE regulatory complex have non-redundant functions in vivo. Curr. Biol. 28, 3674–84.e6. doi: 10.1016/j.cub.2018.10.002, PMID: 30393033 PMC6264382

[ref21] Schneider M. Antes I. (2022). SenseNet, a tool for analysis of protein structure networks obtained from molecular dynamics simulations. PLoS One 17:e0265194. doi: 10.1371/journal.pone.0265194, PMID: 35298511 PMC8929561

[ref22] Torielli L. Guarra F. Shao H. Gestwicki J. E. Serapian S. A. Colombo G. (2025). Pathogenic mutation impairs functional dynamics of Hsp60 in mono- and oligomeric states. Nat. Commun. 16:3158. doi: 10.1038/s41467-025-57958-5, PMID: 40180932 PMC11968893

[ref23] Triveri A. Casali E. Frasnetti E. Doria F. Frigerio F. Cinquini F. . (2023). Conformational behavior of SARS-Cov-2 spike protein variants: evolutionary jumps in sequence reverberate in structural dynamic differences. J. Chem. Theory Comput. 19, 2120–2134. doi: 10.1021/acs.jctc.3c00077, PMID: 36926878 PMC10029694

[ref24] Xie S. Zuo K. De Rubeis S. Bonollo G. Colombo G. Ruggerone P. . (2025b). Impact of genetic variants associated with neurodevelopmental disorders on the WAVE regulatory complex. J. Chem. Inf. Model. 65, 7399–7405. doi: 10.1021/acs.jcim.5c01162, PMID: 40633098 PMC12308791

[ref25] Xie S. Zuo K. De Rubeis S. Ruggerone P. Carloni P. (2025a). Molecular basis of the CYFIP2 and NCKAP1 autism-linked variants in the WAVE regulatory complex. Protein Sci. 34:e5238. doi: 10.1002/pro.5238, PMID: 39660913 PMC11632847

[ref26] Zeidan J. Fombonne E. Scorah J. Ibrahim A. Durkin M. S. Saxena S. . (2022). Global prevalence of autism: a systematic review update. Autism Res. 15, 778–790. doi: 10.1002/aur.2696, PMID: 35238171 PMC9310578

[ref27] Zhao F. Guan W. (2024). Defects of parvalbumin-positive interneurons are implicated in psychiatric disorders. Biochem. Pharmacol. 230:116599. doi: 10.1016/j.bcp.2024.116599, PMID: 39481655

[ref28] Zweier M. Begemann A. McWalter K. Cho M. T. Abela L. Banka S. . (2019). Spatially clustering de novo variants in CYFIP2, encoding the cytoplasmic FMRP interacting protein 2, cause intellectual disability and seizures. Eur. J. Hum. Genet. 27, 747–759. doi: 10.1038/s41431-018-0331-z, PMID: 30664714 PMC6461771

