# Supplementary material for: Common characteristics of variants linked to autism spectrum disorder in the WAVE regulatory complex
Source: Front Comput Neurosci. 2025 Nov 12;19:1704350. doi: 10.3389/fncom.2025.1704350 (PMC12647093; doi:10.3389/fncom.2025.1704350)
Supplement: Supplementary file 1 [file Data_Sheet_1.docx]

Common characteristics of variants linked to autism spectrum disorder in the WAVE regulatory complex

**Song Xie^1,2^, Ke Zuo^1, 3, and 4, *^, Silvia De Rubeis^5, 6, 7, 8, 9, and 10^, Giorgio Bonollo^11^, Giorgio Colombo^11^, Paolo Ruggerone^4,*^, and Paolo Carloni^1, 2, and 12, *^**

^1^Computational Biomedicine, Institute of Neuroscience and Medicine INM-9, Forschungszentrum Jülich GmbH, Jülich, Germany

^2^Department of Physics, RWTH Aachen University, Aachen, Germany

^3^National & Local Joint Engineering Research Center of Targeted and Innovative Therapeutics, Chongqing Key Laboratory of Kinase Modulators as Innovative Medicine, College of Pharmacy (International Academy of Targeted Therapeutics and Innovation), Chongqing University of Arts and Sciences, Chongqing, China

^4^Department of Physics, University of Cagliari, Monserrato, Cagliari, Italy

^5^Seaver Autism Center for Research and Treatment, Icahn School of Medicine at Mount Sinai, New York, USA

^6^Department of Psychiatry, Icahn School of Medicine at Mount Sinai, New York, USA

^7^The Mindich Child Health and Development Institute, Icahn School of Medicine at Mount Sinai, New York, USA

^8^Friedman Brain Institute, Icahn School of Medicine at Mount Sinai, New York, USA

^9^Department of Pharmacological Sciences, Icahn School of Medicine at Mount Sinai, New York, USA

^10^Alper Center for Neural Development and Regeneration, Friedman Brain Institute, Icahn School of Medicine at Mount Sinai, New York, USA

^11^Dipartimento di Chimica, Università di Pavia, Via Taramelli 12, Pavia, Italy

^12^JARA Institute: Molecular Neuroscience and Imaging, Institute of Neuroscience and Medicine INM-11, Forschungszentrum Jülich GmbH, Jülich, Germany

*** Correspondence:**
Ke Zuo, Paolo Ruggerone, and Paolo Carloni
 [k.zuo@stimulate-ejd.eu](mailto:k.zuo@stimulate-ejd.eu), [paolo.ruggerone@unica.it](mailto:paolo.ruggerone@unica.it), [p.carloni@fz-juelich.de](mailto:p.carloni@fz-juelich.de)

**Keywords: WAVE regulatory complex, autism spectrum disorder, missense variants, molecular dynamics, cavity detection**

# Supplementary Methods

## Model Construction

Models of the I664M, E665K, and D724H variants were constructed using SWISS-MODEL (Waterhouse *et al.* 2018) with the reference WT structure, which was obtained from our previous work (Xie *et al.* 2025). Each complex comprised 2,924 residues. Protonation states of Asp, Glu, Arg, Lys, and His were assigned at pH 7.4 with the H++ web server (Gordon *et al.* 2005). The complexes were solvated in orthorhombic water boxes extending at least 13 Å from any solute atom. Na⁺ and Cl⁻ ions were added to neutralize the system and to achieve 150 mM NaCl (**Table S1**). AMBER ff19SB (Tian *et al.* 2020) was used for proteins, OPC for water (Izadi *et al.* 2014), and Li/Merz parameters for Na⁺ and Cl⁻ ions (Li *et al.* 2015).

## Property calculation

Properties were calculated over the final 4.5-µs trajectories from three independent replicates (**Figures S1-S3**), including (i) **Clustering**. MD snapshots were clustered by backbone RMSD using DBSCAN (Ester *et al.* 1996) with 𝜀 = 2.0 Å and min points = 5; (ii) **Radius of gyration**; (iii) **Interfacial area**. For subunits 𝐴 and 𝐵, the interfacial surface area was calculated from solvent-accessible surface areas (SASA; probe radius 1.4 Å) as

$$SASA(A) + SASA(B) - SASA(AB) (1)$$

(iv) **Interfacial contacts.** A contact was defined between two residues if any pair of their non-hydrogen atoms was within 5 Å in a given frame. The residue-pair contact score was computed as the average number of heavy-atom contacts per frame; (v) **Dynamic cross-correlation matrix (DCCM)** (Ichiye *et al.* 1991) and (vi) **principal component vectors** (Amadei *et al.* 1993) were calculated based on Cα atoms; (vii) **H-bond occupancy**. A hydrogen bond was counted when the donor–acceptor distance was ≤ 3.2 Å and the D–H···A angle ≥ 125°. Occupancy was reported as the fraction of frames (in %) in which the criterion was satisfied; (viii) **Distance Fluctuations** ${DF}_{ij}$ (Morra *et al.* 2012). Dynamic coupling between residues 𝑖 and 𝑗 was quantified as the variance of their inter-residue C𝛼 distance around its time average:

${DF}_{ij}=<{(d_{ij}-<d_{ij}>)}^{2}> (2)$

where $d_{ij}$ represents the distance between Cα atoms of the residues in a particular MD frame, and < > denotes averages over a whole trajectory. The normalized ${DF}_{ij}$ value reduces the impact of potential outlier values for pairs in loop regions:

$${NDF}_{ij}=\frac{{tDF}_{ij}}{UB} (3)$$

UB (the upper bound) reads:

$$UB=Q3+ 1.5\times(Q3-Q1) (4)$$

Where Q1 and Q3 are the first and third quartiles of ${DF}_{ij}$ (Rousseeuw *et al.* 2011), respectively.

$t{DF}_{ij}$ is the truncated ${DF}_{ij}$ up to UB:

$$t{DF}_{ij} = {\{}_{{DF}_{ij} if {DF}_{ij} \leq UB}^{UB if {DF}_{ij} \geq UB} (5)$$

The normalized ${DF}_{ij}$score for residue i is obtained by averaging over the ${NDF}_{ij}$

$${NDF}_{i}= \frac{1}{2924}\cdot(\sum_{1}^{2924} {NDF}_{ij}) (6)$$

The difference in ${NDF}_{i}$score (Δ${NDF}_{i}$, hereafter) between WT and WRC variants

$$\Delta{NDF}_{i} = {NDF}_{i}^{variant} -{NDF}_{i}^{WT} (7)$$

correlates with the efficiency of the folding/unfolding transitions: the higher the values, the more efficient the transitions in the residues involved (Rehn *et al.* 2016; Moroni *et al.* 2018; Triveri *et al.* 2023; Castelli *et al.* 2024; Torielli *et al.* 2025); (ix) **Allosteric score** $\boldsymbol{AS}_{\boldsymbol{i}}$ (Schneider *et al.* 2022). This property quantifies the allosteric importance of residue 𝑖 within the complex. Each system is presented as a residue-residue interaction network. Nodes represent amino-acid residues, and edges connect residue pairs that interact. Edge types were assigned for van der Waals contacts (heavy-atom distance ≤ 5 Å) and hydrogen bonds, allowing multiple edges between a pair when multiple interaction types are present. ${AS}_{i}$ values were then computed on these networks:

$${AS}_{i} = ({NBC}_{i} +{NNCF}_{i})/2 (8)$$

Where 𝑁𝐵𝐶_𝑖_ and 𝑁𝑁𝐶𝐹_𝑖_ denote the normalized betweenness centrality and normalized node correlation factor of residue *i*, respectively. BC is widely used to detect functional residues in protein structure networks, as residues critical for signal transfer lie on many shortest paths. To mitigate noise from transient contacts in MD trajectories, we retained only residue–residue edges with a contact present in >50% of the simulations when computing ${NBC}_{i}$ (Schneider *et al.* 2022). NCF quantifies the allosteric importance of a residue by measuring how strongly its conformational dynamics correlate with those of its local neighborhood (Schneider *et al.* 2022). Unlike global path–based measures such as BC, NCF down-weights spurious interactions through its reliance on regional connectivity and mutual-information-based correlations; therefore, all detected network edges were used for ${NNCF}_{i}$ (Schneider *et al.* 2022). The change in allosteric score between WT and a variant was summarized as,

$$\Delta{AS}_{i} = {AS}_{i}^{variant} -{AS}_{i}^{WT} (9)$$

$\Delta{AS}_{i}$ reports how a mutation alters the allosteric importance of residue i. Positive $\Delta{AS}_{i}$ values indicate increased allosteric importance, while negative values indicate the opposite (Schneider *et al.* 2022).

Analyses of (i)–(vii) were performed with CPPTRAJ(Roe *et al.* 2013), and (viii) and (ix) were calculated by distance_fluctuation.py codes (Morra *et al.* 2012), and SenseNet (Schneider *et al.* 2022), respectively. Trajectories for analysis of the WT, R87C, A455P, and Q725R WRC were obtained from our previous work(Xie *et al.* 2025).

# Supplementary Figures

**
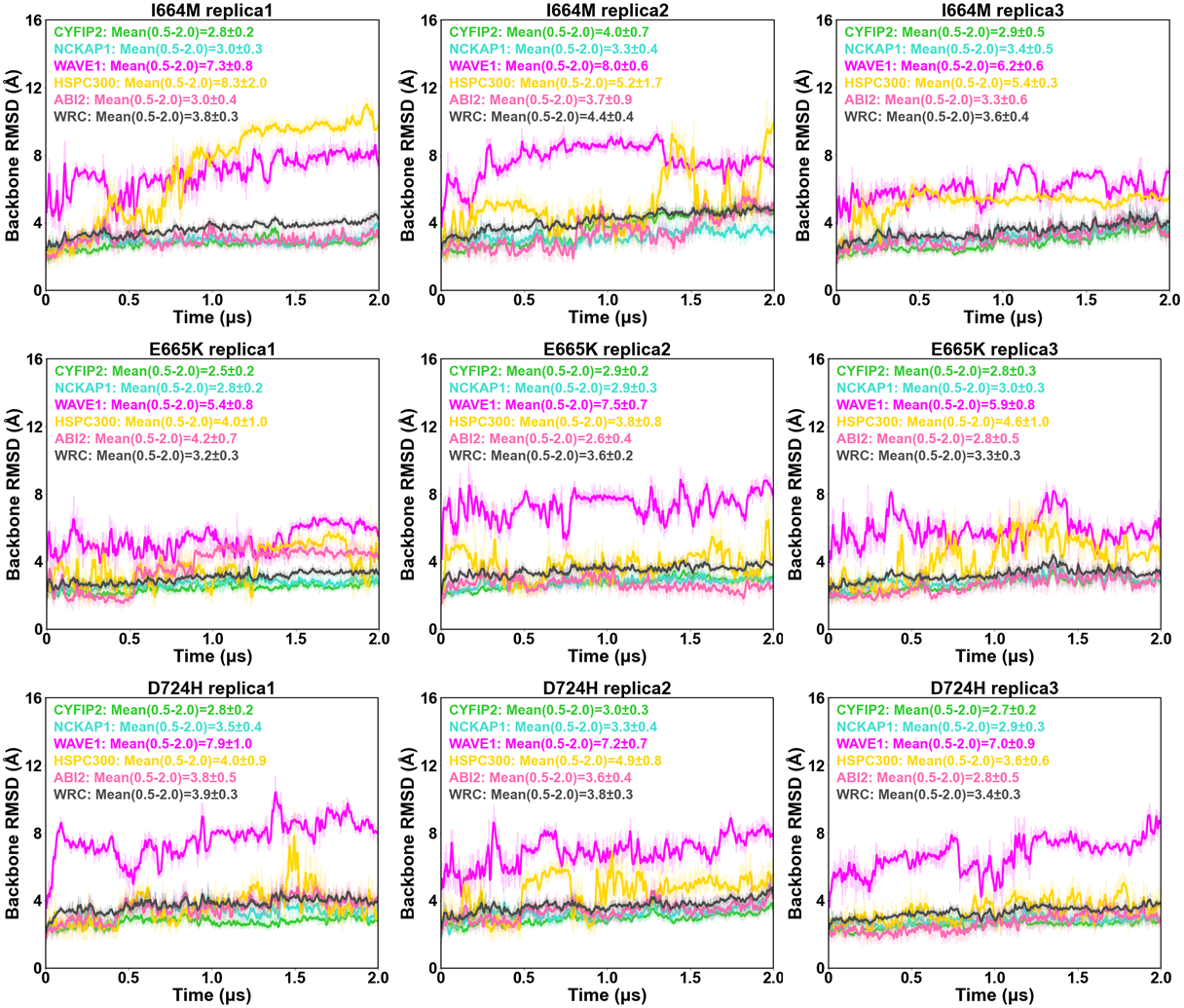
**

**Figure S1. Backbone root-mean-square deviation (RMSD) of ASD-linked variants (I664M, E665K, and D724H) plotted as a function of simulated time.** Most RMSD values show fluctuations < 0.5 Å during the final 1.5 µs. As expected, the RMSD values for the WAVE1 and HSPC300 flexible loops exhibit relatively large fluctuations in specific regions (**Figures S2 and S3**). In contrast, the folded regions exhibit RMSD fluctuations of 0.6 Å or less, except for replica 1 in D724H (see **Figures S2** and **S3**).

**
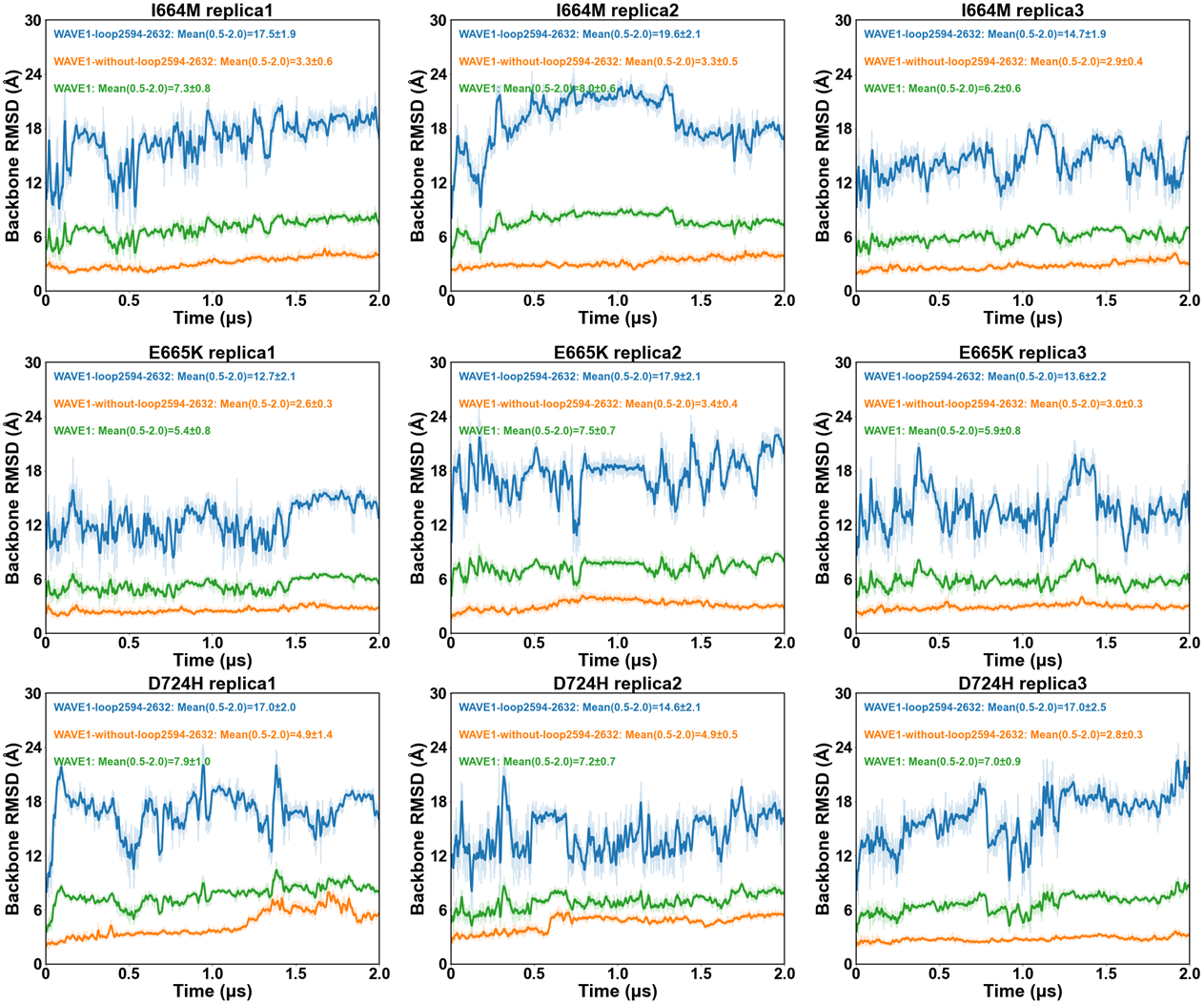
**

**Figure S2. Backbone RMSD**  of the WAVE1 protein versus simulation time.

**
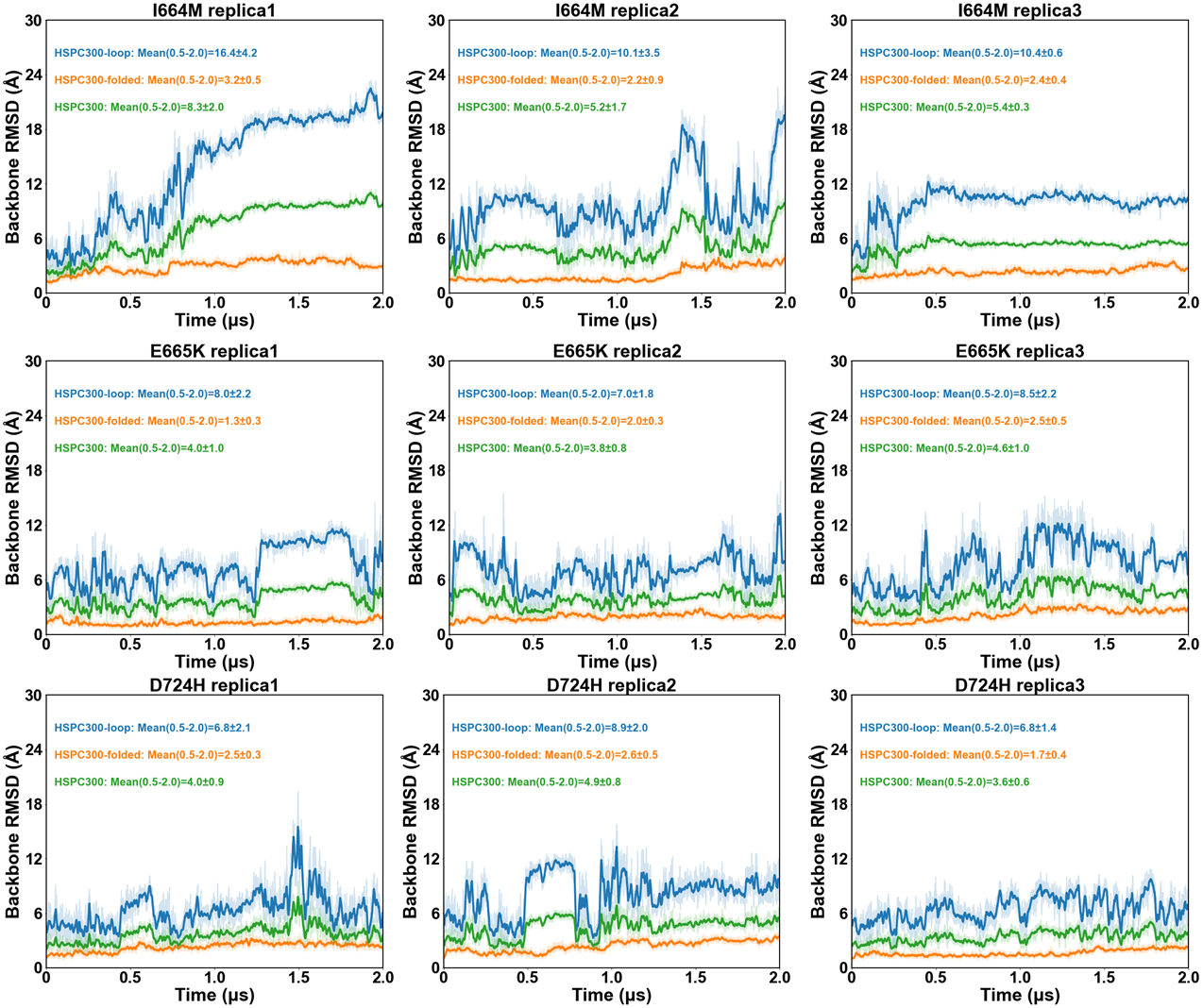
**

**Figure S3. Backbone RMSD** of the HSPC300 protein versus simulation time.


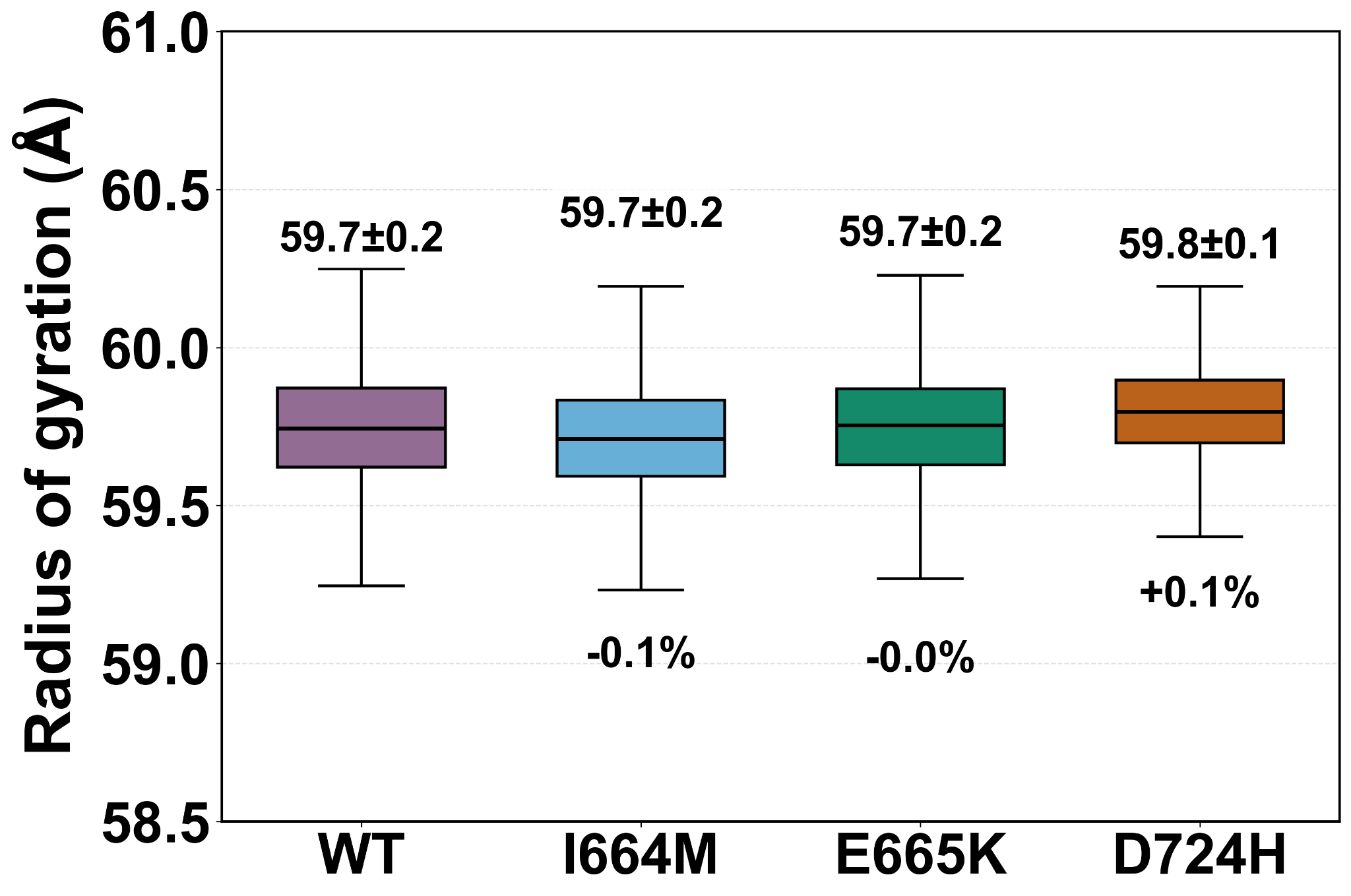


**Figure S4. The radius of gyration of the WT, I664M, E665K, and D724H WRC.** The changes in the variants relative to the WT are labeled. Trajectories for analysis of the WT complex were obtained from previous work (Xie *et al.* 2025).


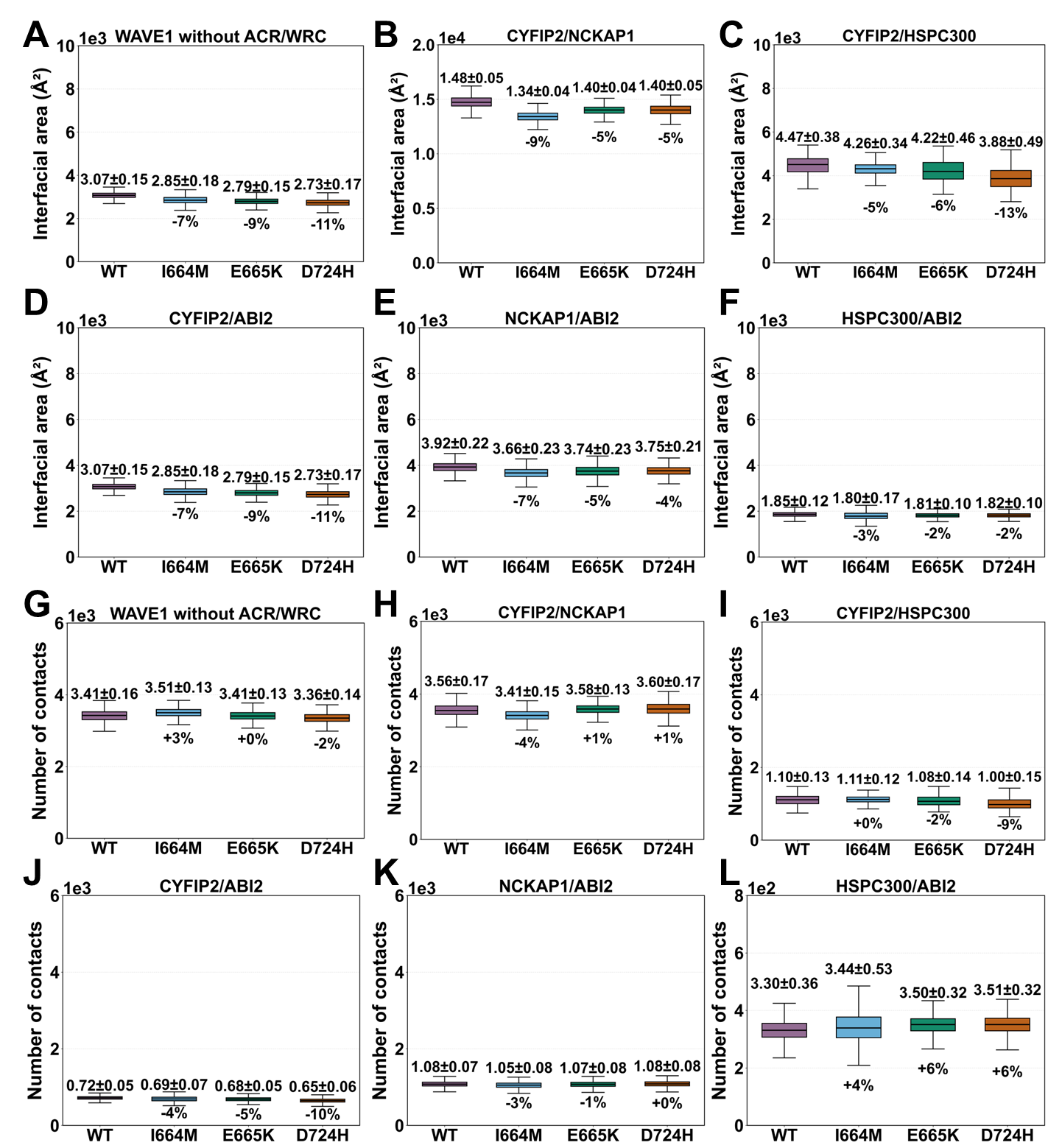


**Figure S5.** **Interfacial areas (A-F) and number of heavy-atom contacts (G-L) of all protein/protein interfaces except for the ACR/WRC interface, which is shown in Figures 3A and 3B.** The changes in the variants relative to the WT are labeled. The data for the WT WRC are obtained from previous work (Xie *et al.* 2025).


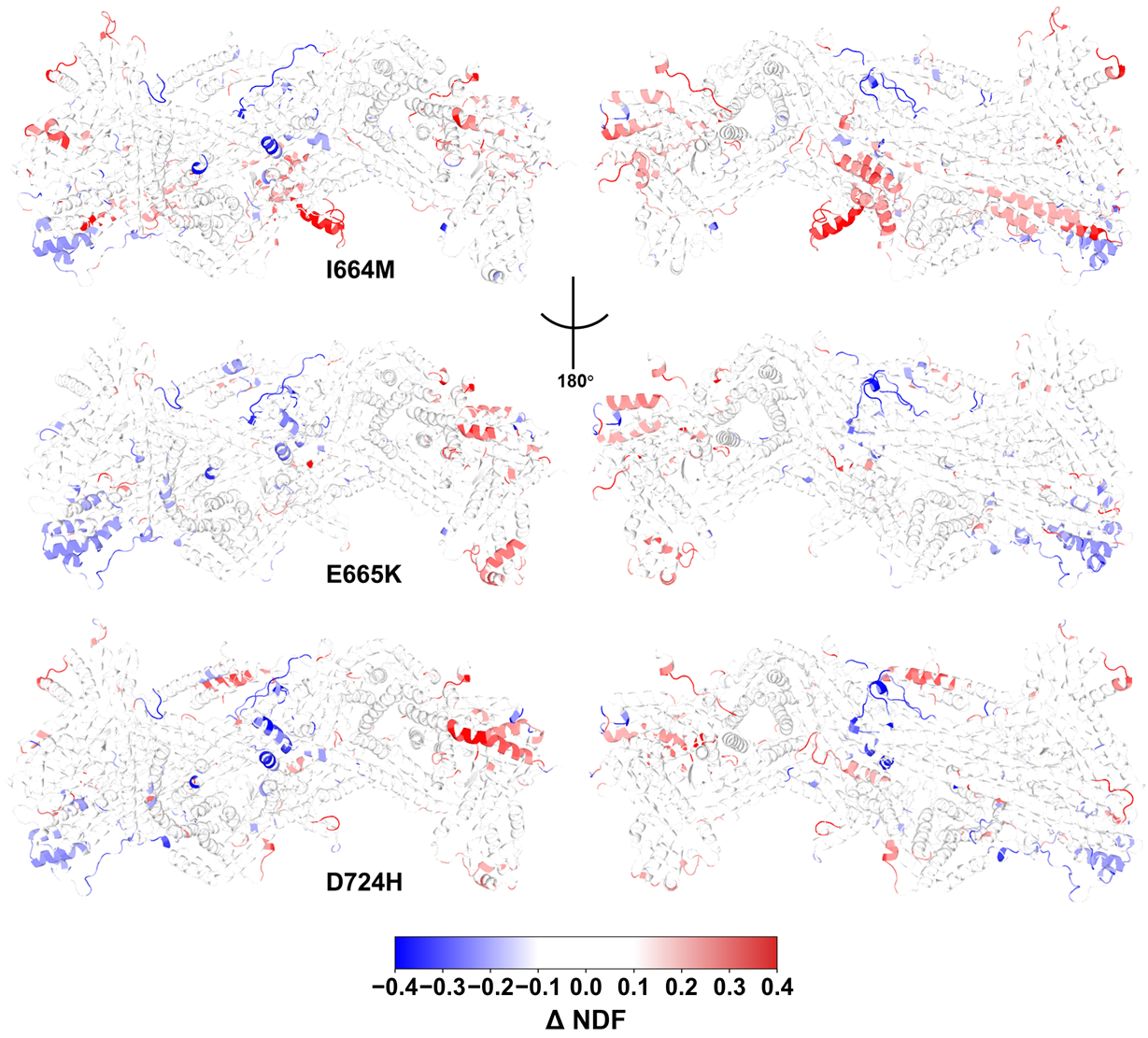


**Figure S6.** **The Δ*NDF_i_* values (i=1, 2, … 2924) for the four systems range from −0.4 (blue) to +0.4 (red).** The proteins are shown as cartoons.


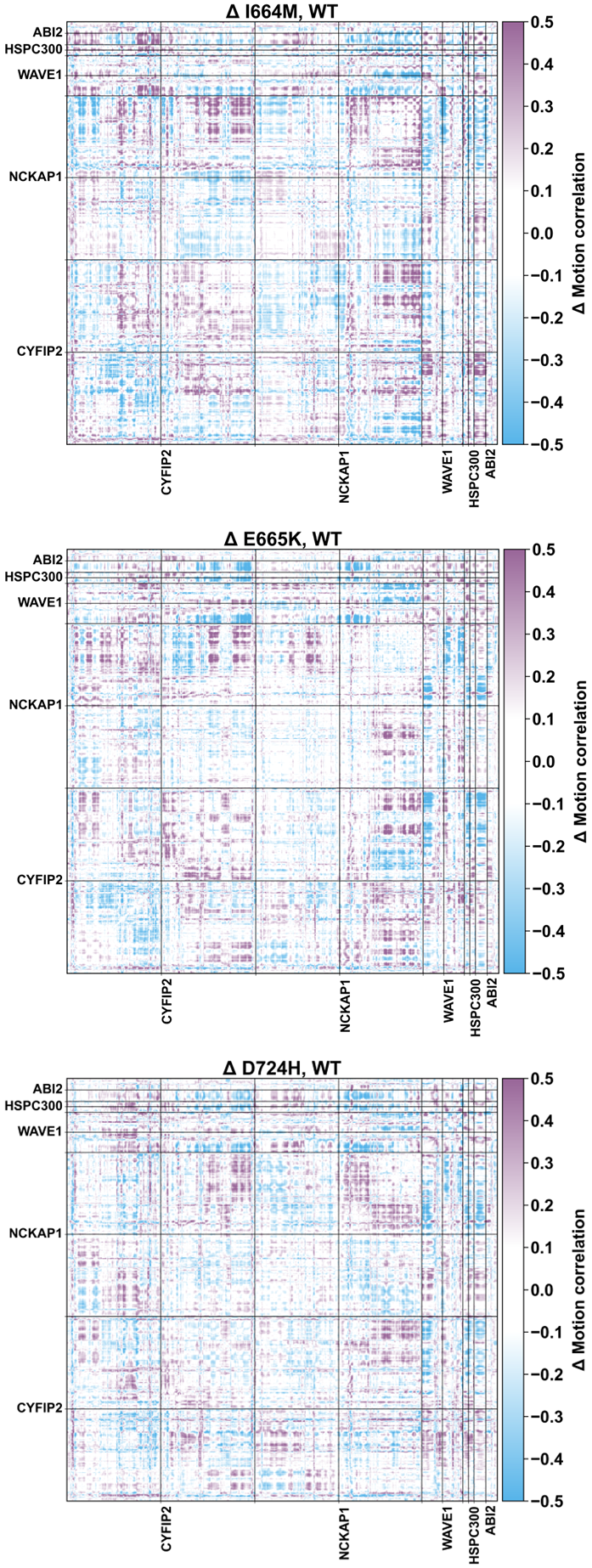


**Figure S7.** **The effect of variants on the motion correlation.** The values range from −0.5 (blue) to +0.5 (purple).


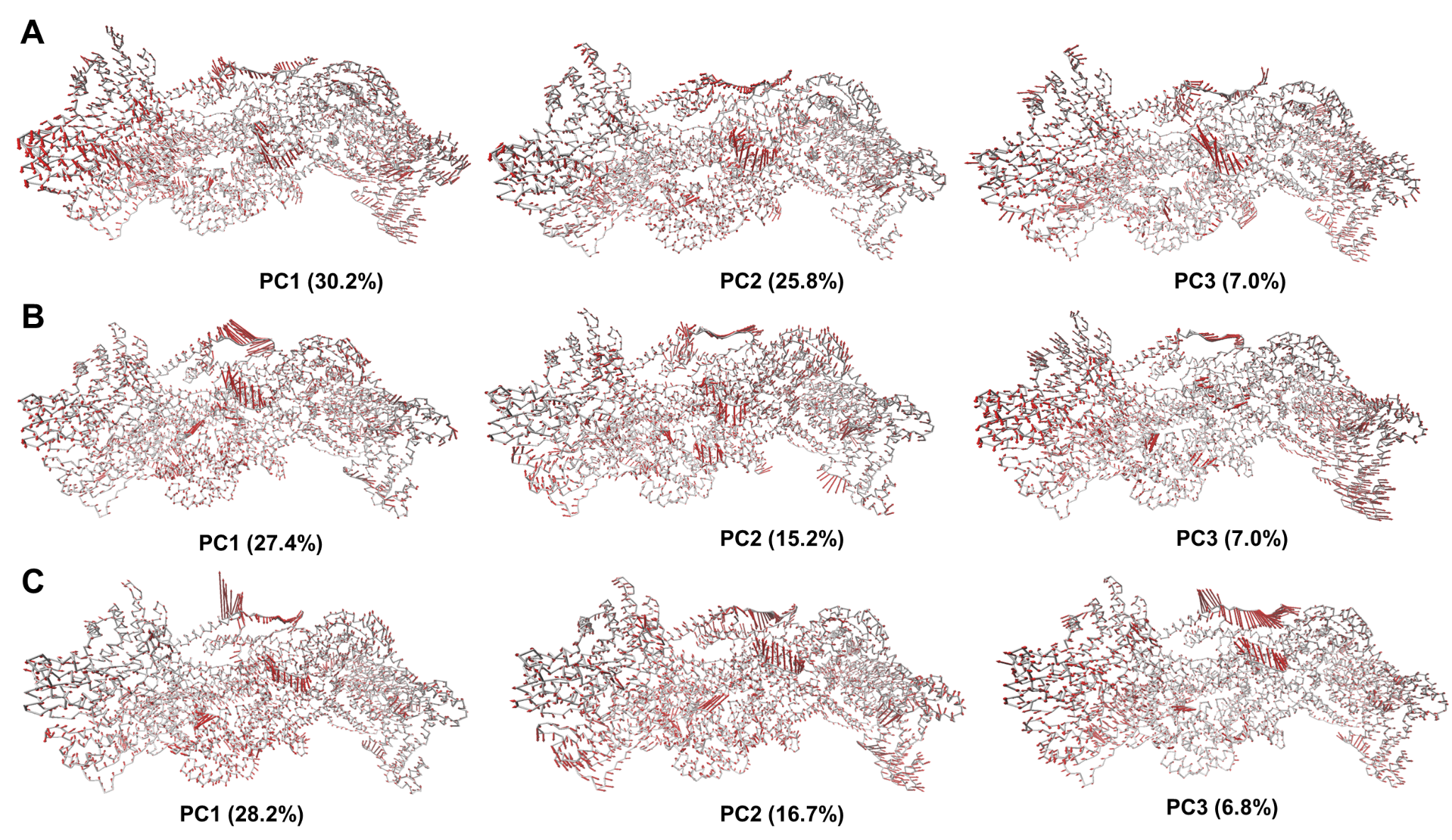


**Figure S8**. (A-C) The largest three eigenvectors (PC1–PC3) from our principal component analysis on the three variants (I664M, E665K, and D724H). The percentage of total motion variance by each eigenvector is given in parentheses. The protein is displayed as a white ribbon; the direction of motion is shown as a red arrow.

# Supplementary Tables

**Table S1.** Information about simulated systems.

| **System** | **Number of Na^+^** | **Number of Cl^−^** | **Number of water molecules** |
| --- | --- | --- | --- |
| I664M WRC | 399 | 382 | 106,159 |
| E665K WRC | 397 | 382 | 106,168 |
| D724H WRC | 398 | 382 | 106,567 |

**Table S2.** Occupancy of the H-bonds and contact scores formed by residues undergoing mutations in the four systems investigated here. The data for the WT complex were obtained from previous work (Xie *et al.* 2025).

| Residue pairs | WT | | I664M | | E665K | | D724H |
| --- | --- | --- | --- | --- | --- | --- | --- |
|  | Salt-bridge/H-bond occupancy | | | | | | |
| E665(CYFIP2)/K164(ACR) or K665(CYFIP2)/K164(ACR) | 53% | | 55% | | 0% | | 46% |
| D724(CYFIP2)/Q110(WAVE1) or H724(CYFIP2)/Q110(WAVE1) | 27% | | 29% | | 27% | | 6% |
|  | Contact scores | | | | | | |
| I664(CYFIP2)/F157(ACR) or M664(CYFIP2)/F157(ACR) | 6 (99%) | 5 (96%) | | 3 (73%) | | 5 (88%) | |
| I664(CYFIP2)/W161(ACR) or M664(CYFIP2)/W161(ACR) | 2 (94%) | 2 (54%) | | 2 (94%) | | 1 (76%) | |
| D724(CYFIP2)/V531(ACR) or H724(CYFIP2)/V531(ACR) | 12 (100%) | 1 (100%) | | 12 (100%) | | 12 (100%) | |
| D724(CYFIP2)/L111(WAVE1 without ACR) or H724(CYFIP2)/L111(WAVE1 without ACR) | 1 (50%) | 1 (55%) | | 1 (46%) | | 5 (71%) | |

**Table S3**. Cosine similarity (Salton *et al.* 1975; Connor 2016) among the different motion correlation matrices when comparing variants with the WT. The data for the R87C, A455P, and Q725R were obtained from previous work (Xie *et al.* 2025).

|  | **R87C** | **A455P** | **I664M** | **E665K** | **D724H** | **Q725R** |
| --- | --- | --- | --- | --- | --- | --- |
| **R87C** | 1 | 0.55 | 0.55 | 0.58 | 0.50 | 0.56 |
| **A455P** | 0.55 | 1 | 0.43 | 0.63 | 0.48 | 0.48 |
| **I664M** | 0.55 | 0.43 | 1 | 0.46 | 0.44 | 0.49 |
| **E665K** | 0.58 | 0.63 | 0.46 | 1 | 0.58 | 0.54 |
| **D724H** | 0.50 | 0.48 | 0.44 | 0.58 | 1 | 0.53 |
| **Q725R** | 0.56 | 0.48 | 0.49 | 0.54 | 0.53 | 1 |

**Table S4**. Spearman correlation (Sedgwick 2014) among the different motion correlation matrices on passing from variants to the WT. The data for the R87C, A455P, and Q725R were obtained from previous work (Xie *et al.* 2025).

|  | **R87C** | **A455P** | **I664M** | **E665K** | **D724H** | **Q725R** |
| --- | --- | --- | --- | --- | --- | --- |
| **R87C** | 1 | 0.50 | 0.52 | 0.55 | 0.46 | 0.53 |
| **A455P** | 0.50 | 1 | 0.38 | 0.60 | 0.45 | 0.44 |
| **I664M** | 0.52 | 0.38 | 1 | 0.42 | 0.39 | 0.46 |
| **E665K** | 0.55 | 0.60 | 0.42 | 1 | 0.55 | 0.51 |
| **D724H** | 0.46 | 0.45 | 0.39 | 0.55 | 1 | 0.50 |
| **Q725R** | 0.53 | 0.44 | 0.46 | 0.51 | 0.50 | 1 |

**Table S5**. Summary of the impact of six ASD-linked variants on the structural dynamics of WRC.

|  | **Location** | **Impact on global unfolding or loss of complex stability** | **Impact on ACR/WRC interactions** | **Impact on long-range allosteric communication within the complex** | **Impact on V-helix stability** | **Impact on ACR large-scale movement within the complex** |
| --- | --- | --- | --- | --- | --- | --- |
| **R87C** | ACR/CYFIP2 interface | No | Decrease | Decrease | Decrease | Increase |
| **A455P** | CYFIP2 interior | No | Decrease | Decrease | Decrease | Increase |
| **I664M** | ACR/CYFIP2 interface | No | Decrease | Decrease | Mixed (both increase and decrease) | Decrease |
| **E665K** | ACR/CYFIP2 interface | No | Decrease | Decrease | Decrease | Increase |
| **D724H** | ACR/CYFIP2 interface | No | Decrease | Decrease | Decrease | Increase |
| **Q725R** | ACR/CYFIP2 interface | No | Decrease | Decrease | Decrease | Increase |

# Reference

Amadei, A., Linssen, A. B. M., Berendsen, H. J. C. (1993). Essential dynamics of proteins. *Proteins: Structure, Function, and Bioinformatics* 17, 412-25, doi: 10.1002/prot.340170408

Castelli, M., Magni, A., Bonollo, G., Pavoni, S., Frigerio, F., Oliveira, A. S. F.*, et al.* (2024). Molecular mechanisms of chaperone-directed protein folding: Insights from atomistic simulations. *Protein Science* 33, e4880, doi: 10.1002/pro.4880

Connor, R. A tale of four metrics. Cham. Pp 210-7. Springer International Publishing, 2016.

Ester, M., Kriegel, H.-P., Sander, J., Xu, X. A density-based algorithm for discovering clusters in large spatial databases with noise. In: Proceedings of the second international conference on knowledge discovery and data mining. Pp 226–31. (AAAI Press, Portland, Oregon) 1996. 10.5555/3001460.3001507

Gordon, J. C., Myers, J. B., Folta, T., Shoja, V., Heath, L. S., Onufriev, A. (2005). H++: a server for estimating p Ka s and adding missing hydrogens to macromolecules. *Nucleic Acids Research* 33, W368-W71, doi: 10.1093/nar/gki464

Ichiye, T., Karplus, M. (1991). Collective motions in proteins: A covariance analysis of atomic fluctuations in molecular dynamics and normal mode simulations. *Proteins: Structure, Function, and Bioinformatics* 11, 205-17, doi: 10.1002/prot.340110305

Izadi, S., Anandakrishnan, R., Onufriev, A. V. (2014). Building water models: A different approach. *The Journal of Physical Chemistry Letters* 5, 3863-71, doi: 10.1021/jz501780a

Li, P., Song, L. F., Merz, K. J. (2015). Systematic parameterization of monovalent Ions employing the nonbonded model. *Journal of Chemical Theory and Computation* 11, 1645-57, doi: 10.1021/ct500918t

Moroni, E., Agard, D. A., Colombo, G. (2018). The structural asymmetry of mitochondrial Hsp90 (Trap1) determines fine tuning of functional dynamics. *Journal of Chemical Theory and Computation* 14, 1033-44, doi: 10.1021/acs.jctc.7b00766

Morra, G., Potestio, R., Micheletti, C., Colombo, G. (2012). Corresponding functional dynamics across the Hsp90 chaperone family: Insights from a multiscale analysis of MD simulations. *PLOS Computational Biology* 8, e1002433, doi: 10.1371/journal.pcbi.1002433

Rehn, A., Moroni, E., Zierer, B. K., Tippel, F., Morra, G., John, C.*, et al.* (2016). Allosteric regulation points control the conformational dynamics of the molecular chaperone Hsp90. *Journal of Molecular Biology* 428, 4559-71, doi: 10.1016/j.jmb.2016.09.014

Roe, D. R., Cheatham, T. E., III. (2013). PTRAJ and CPPTRAJ: Software for processing and analysis of molecular dynamics trajectory data. *Journal of Chemical Theory and Computation* 9, 3084-95, doi: 10.1021/ct400341p

Rousseeuw, P. J., Hubert, M. (2011). Robust statistics for outlier detection. *WIREs Data Mining and Knowledge Discovery* 1, 73-9, doi: 10.1002/widm.2

Salton, G., Wong, A., Yang, C.-S. (1975). A vector space model for automatic indexing. *Commun. ACM* 18, 613-20, doi: 10.1145/361219.361220

Schneider, M., Antes, I. (2022). SenseNet, a tool for analysis of protein structure networks obtained from molecular dynamics simulations. *PLOS ONE* 17, e0265194, doi: 10.1371/journal.pone.0265194

Sedgwick, P. (2014). Spearman’s rank correlation coefficient. *BMJ* 349, g7327, doi: 10.1136/bmj.g7327

Tian, C., Kasavajhala, K., Belfon, K. A. A., Raguette, L., Huang, H., Migues, A. N.*, et al.* (2020). ff19SB: Amino-acid-specific protein backbone parameters trained against quantum mechanics energy surfaces in solution. *Journal of Chemical Theory and Computation* 16, 528-52, doi: 10.1021/acs.jctc.9b00591

Torielli, L., Guarra, F., Shao, H., Gestwicki, J. E., Serapian, S. A., Colombo, G. (2025). Pathogenic mutation impairs functional dynamics of Hsp60 in mono- and oligomeric states. *Nature Communications* 16, 3158, doi: 10.1038/s41467-025-57958-5

Triveri, A., Casali, E., Frasnetti, E., Doria, F., Frigerio, F., Cinquini, F.*, et al.* (2023). Conformational behavior of SARS-Cov-2 spike protein variants: evolutionary jumps in sequence reverberate in structural dynamic differences. *Journal of Chemical Theory and Computation* 19, 2120-34, doi: 10.1021/acs.jctc.3c00077

Waterhouse, A., Bertoni, M., Bienert, S., Studer, G., Tauriello, G., Gumienny, R.*, et al.* (2018). SWISS-MODEL: homology modelling of protein structures and complexes. *Nucleic Acids Research* 46, W296-W303, doi: 10.1093/nar/gky427

Xie, S., Zuo, K., De Rubeis, S., Bonollo, G., Colombo, G., Ruggerone, P.*, et al.* (2025). Impact of genetic variants associated with neurodevelopmental disorders on the WAVE regulatory complex. *Journal of Chemical Information and Modeling* 65, 7399-405, doi: 10.1021/acs.jcim.5c01162
